# The Ethics of Germline Gene Editing

**DOI:** 10.1111/japp.12249

**Published:** 2016-11-09

**Authors:** Christopher Gyngell, Thomas Douglas, Julian Savulescu

**Affiliations:** ^1^ Oxford Uehiro Centre for Practical Ethics University of Oxford Littlegate House St Ebbes Street Oxford OX1 1PT UK; ^2^ Oxford Uehiro Centre for Practical Ethics and Mansfield College University of Oxford; ^3^ Oxford Uehiro Centre for Practical Ethics University of Oxford & Visiting Fellow Faculty of Law Queensland University of Technology

## Abstract

Germline Gene Editing (GGE) has enormous potential both as a research tool and a therapeutic intervention. While other types of gene editing are relatively uncontroversial, GGE has been strongly resisted. In this article, we analyse the ethical arguments for and against pursuing GGE by allowing and funding its development. We argue there is a strong case for pursuing GGE for the prevention of disease. We then examine objections that have been raised against pursuing GGE and argue that these fail. We conclude that the moral case in favour of pursuing GGE is stronger than the case against. This suggests that pursuing GGE is morally permissible and indeed morally desirable.

## 1 Introduction

In April 2015, it was announced that gene editing techniques had been used to modify the DNA sequences of human embryos for the first time.^1^ The study by Liang and co‐authors attempted to use the gene editing technique CRISPR to reverse the genetic mutations that lead to the disease muscular dystrophy. The study marked the first time the human germline – the DNA individuals pass to their children and subsequent descendants – had been intentionally modified.^2^

The rise of gene editing technologies has been rapid. While crude genetic engineering technologies have been available for over two decades, early techniques did not have serious potential as clinically useful modifiers of human DNA. They relied on viruses to deliver novel genetic material to the cell. This often only changed one of the two copies of the target gene, meaning animals had to be bred together to make modifications effective. This method also made unintended changes to large segments of the genome, and only a small proportion of the modified animals did not suffer serious side effects.^3^

The development of techniques that use engineered enzymes, rather than viruses, to alter DNA has revolutionised genetic engineering. These techniques were given the collective moniker ‘gene editing’ to reflect their increased efficiency and precision over older methods. Gene editing techniques have been used to make precise changes to the genes of yeast, fish, plants, rodents, pigs, and primates. In a recent study, gene editing was used to inactivate 62 genes of a retrovirus in a pig cell line, a significant step towards generating pig organs suitable for xenotransplantation.^4^ In October 2015, researchers created a beagle with double the normal muscle mass by editing a gene involved in muscle growth.^5^

Despite these far‐reaching applications, public debate has focused on the ethics of human germline gene editing (GGE).^6^ Some scientists and public interest groups, including the United Nations Educational Scientific and Cultural Organization (UNESCO) have called for an international ban on any gene editing research in human embryos. The US‐based National Institutes of Health maintained that performing such research would cross ‘a line that should not be crossed’. Other scientists have taken a more moderate view. In 2015, an international summit co‐hosted by the US National Academy of Sciences and US National Academy of Medicine, the UK Royal Society, and the Chinese Academy of Sciences rejected calls for a ban on GGE in research. Instead they argued for a temporary ban on the clinical use of GGE that should be regularly revisited ‘as scientific knowledge advances and societal views evolve.’^7^ Similarly, the Hinxton Group, an international consortium of scientists, ethicists and policy experts called for the continuation of GGE in basic research and a pause on any reproductive applications.^8^

In this article, we examine the ethics of pursuing GGE, where ‘pursuing’ GGE would entail at least (i) allowing scientists significant freedom to conduct GGE research, and (ii) the investment of significant public resources in such research. We begin, in Section 2, by outlining the case for pursuing GGE, both for the direct prevention of disease, and as a research tool. In Section 3, we analyse some of the ethical objections that have been raised against pursuing GGE. We argue that all of these objections fail. We conclude that the moral case in favour of pursuing GGE is stronger than the case against and that pursuing GGE is thus morally permissible and morally desirable.

## 2 The Case for Pursuing GGE

### 2.1 The Medical Case

If proven acceptably safe, the most obvious clinical application of GGE will be to prevent genetic disease. Roughly 6% of all babies born have a serious birth defect of genetic or partly genetic origin.^9^ If we can identify the genes responsible for these conditions, it will in principle be possible to prevent these conditions through GGE.

Many believe that GGE is unnecessary for this goal. Couples wishing to avoid genetic disease in their children can use in‐vitro fertilisation (IVF) to create multiple embryos, all of which can be tested for genetic disease before implantation. IVF and pre‐implantation genetic diagnosis (PGD) are already widely used to avoid passing on over 250 genetic diseases including cystic fibrosis, Huntington's disease, haemophilia and phenylketonuria.

Marcy Darnovsky, the executive director of the non‐profit organisation, The Center for Genetics and Society, has claimed that ‘there is no persuasive medical reason to manipulate the human germline because inherited genetic diseases can be prevented using embryo screening techniques, among other means’.^10^ This view was also expressed in a *Nature* commentary calling for a moratorium on GGE; the authors stated that we ‘cannot imagine a situation in which its use in human embryos would offer a therapeutic benefit over existing and developing methods.’^11^

However, there is in fact a significant medical case for pursuing GGE, which we will now outline. In doing so, and throughout, we assume that current people have moral reasons to prevent the occurrence of disease in future people. Moreover, we assume that these reasons apply regardless of whether our current actions to prevent disease make any future people better off than they would otherwise have been.

This assumption is consistent with standard views regarding, for example, the desirability of eradicating infectious diseases, such as malaria and smallpox, and of adopting policies that reduce smoking and alcohol use during pregnancy. Most would agree that there are reasons to adopt such measures and policies, even if they will not affect anyone who currently exists. These reasons are best understood as reasons to prevent the occurrence of disease in future people. Moreover, most would accept that there are reasons to adopt such measures and policies even if doing so will affect no currently existing person *and affect which future people exist*, so that they make no future person better off than they would otherwise have been.

#### 2.1.1 Single Gene Disorders

For some couples, using GGE may be the only way to avoid passing on single gene disorders. Approximately 19% of women undergoing IVF only produce one viable embryo.^12^ Imagine that two carriers of the gene for cystic fibrosis (CF) wish to have a child together. They have a one in four chance of having a child with CF. They use IVF because they want to avoid this outcome. However, they only produce one embryo and this has two copies of the CF gene and will thus develop CF. In cases such as this, selection is not an option; however, GGE could be used to prevent CF.

Even when couples produce more than one embryo, in some cases selection will not avoid disease. In late onset dominant conditions, like Huntington's disease, some patients carry two copies of the disease‐causing gene. This means that every embryo produced from their gametes will be predisposed to disease. GGE will be the only way such individuals could have children that are biologically related to them and who are not predisposed to disease.

Therefore, a straightforward medical application of GGE is to allow single gene disorders to be avoided in cases where selection is not possible.

Some might object that such cases are very rare. Yet even if GGE would only enable a small number of additional people to avoid passing genetic diseases onto their children, this still generates a reason to pursue it; recall, we are assuming that we have reasons to prevent disease in future people.

Moreover, even in cases where IVF and PGD can be used to avoid genetic disease in a couple's offspring, GGE may have the advantage of preventing disease in subsequent generations as well. In the case of autosomal recessive disorders, children who are born as the result of PGD are often carriers of the condition their parents selected against. These individuals have a greater‐than‐normal risk of themselves having children with the disease.

Imagine a couple who both carry the gene for muscular dystrophy (MD), and wish to avoid passing the disease on to their children. If these parents use GGE, all disease‐causing genes can be removed from an embryo. In the absence of spontaneous mutations, this will make it impossible for their children, and their grandchildren, to develop MD. Even though genetic selection could in theory be used to select a non‐carrier, this could require creation of a large number of embryos, which may not be possible. If they use PGD, their child may still be a carrier of the MD gene, and their grandchildren will still be at risk of developing MD. This couple might reasonably prefer GGE to PGD, and it is plausible that we have reasons to enable the fulfilment of such reasonable preferences, for example, by pursuing GGE.

Moreover, even leaving aside prospective parents’ preferences, cases such as this suggest that there is reason to pursue GGE. Using GGE to remove all disease‐causing genes from an embryo will lower the total frequency of disease‐causing genes in the gene pool, and therefore the incidence of such diseases in future generations. And this, on the assumption we introduced above, is an outcome that we have reason to bring about.

Finally, genetic selection replaces one individual with a disease with a healthy individual. It does not benefit those with disease. Its benefits are impersonal. GGE on the other hand could provide benefits to individuals who would otherwise be born with genetic disorders – it could cure their disorders.^13^ In cases where the embryo will in any case be brought to term, and in the absence of GGE would be afflicted by disease, its benefits are arguably person‐affecting. It is plausible that person‐affecting benefits are more important than impersonal benefits.^14^

These considerations show that, were GGE developed to the point that it could be used to avoid single gene disorders, there would be strong reasons to use it in some cases. GGE would be a more widely applicable intervention than PGD, as it would allow people to avoid genetic diseases in cases where selection is not possible. It would also be a more effective intervention insofar as it would allow families to remove all disease‐causing genes from their lineages, minimising the risk of disease in their decedents and future generations. And it has the potential to produce person‐affecting benefits.

#### 2.1.2 Other Disorders

Most common diseases are not the result of single gene mutations. They are the result of a polygenic disposition together with environmental influences. For example, genome wide association studies have identified at least 44 genes involved in diabetes;^15^ 35 genes involved in coronary artery disease;^16^ and over 300 genes involved in common cancers.^17^

Traditional selection methods, like IVF and PGD, are not powerful enough to select against polygenic diseases.^18^ Say 20 genes contribute to a particular trait. If a couple wish to use PGD to select for 20 different genes in an embryo, they would need to create around 10,000 embryos to make it sufficiently likely that one will have the right combination at all 20 loci. During routine IVF, couples rarely produce more than 10 embryos. This would give them less than a 1% chance of being able to select an embryo with the desired genotype.

GGE allows multiple changes to be made to a single embryo, and could therefore target many different genes simultaneously. It has already been used to make more than 60 simultaneous changes in animal embryos, and this number can be expected to increase. This ability could make GGE a powerful disease‐preventing technology. Three out of every ten deaths in those under 70 are caused by chronic diseases, like cancer, diabetes and heart disease. GGE could be a powerful tool in the fight against these diseases and could ensure that those treated with gene editing have the best chance to live healthily into old age.

We currently do not understand the genetics underlying common polygenic diseases well enough to consider using GGE to reduce their incidence. But it is clear such applications are a long‐term possibility.

Some might object that as polygenic disorders have environmental causes as well as genetic ones, GGE is unnecessary to prevent them. Even if we determine the genetic changes needed to prevent or reduce the risk of these disorders, we could more easily use environmental interventions. But it does not follow from the fact that a disease is partly or even primarily caused by environmental factors that environmental interventions are effective in preventing it, let alone the most effective means of prevention. The environmental causes might be more difficult to identify or alter than the genetic causes, and even when we can identify the environmental causes, polygenic disorders may be optimally prevented by biological interventions. For example, using GGE techniques to make the immune system more efficient at destroying cancer cells might be the best way to reduce the incidence of cancer, even if cancer is predominantly caused by environmental factors. Indeed, the fact that polygenic disorders currently result in so many premature deaths, despite the fact we have identified many of their environmental risk factors, indicates we need to explore novel approaches to their treatment.

In addition, in most cases we should not think of environmental and genetic prevention techniques as mutually exclusive alternatives. Even where environmental interventions could be somewhat effective in preventing a disease, they are unlikely to reduce the risk of disease to zero, suggesting that there will still be a case for deploying genetic interventions alongside environmental ones.

Another speculative long‐term medical benefit of GGE is spreading resistance to infectious diseases.^19^ Some people have genes that make them resistant to particular pathogens. In cases of infectious disease outbreaks, GGE could ensure that everyone born in a region is resistant to local pathogens. Imagine an Ebola outbreak that surpasses traditional means of containment. As genetic background determines susceptibility to Ebola,^20^ genes that make individuals highly resistant to Ebola may soon be identified. In such a case, GGE could be used to spread resistance genes in a population, effectively immunising the next generation.

Such actions will be highly impractical if GGE could only be performed through IVF. But in the future it may be possible to perform ‘in vivo’ germline gene edits. In vivo gene edits of muscle stem cells have been performed in mice,^21^ indicating that in vivo edits of spermatogonial stem cells (SSCs) in humans may be feasible in the future. Using gene editing on SSC's in the testes could modify all the sperm that a male produces, thus ensuring they pass on particular changes to all of their descendants. This could allow resistance genes to spread quickly through populations without the need for IVF.

All of the benefits discussed in this section (2.1.2) are highly speculative. However, given the large disease burden caused by infectious and polygenetic diseases, the potential benefits are highly significant. They add further weight to the medical case of pursuing GGE.

### 2.2 The Research Case

Even if the medical case in favour of pursuing GGE fails, it would not follow that all GGE should be banned, or that it should be deprived of significant public resources. GGE has an important role in basic research which should be distinguished from its potential applications in disease prevention.

Many jurisdictions permit and fund embryo research that seeks to further our understanding of early development, and GGE could have a unique and beneficial role to play in this research. Using gene editing techniques, researchers can investigate the role of genetics in human development. Indeed, this was the motivation of the researchers who lodged the first application to perform GGE in the United Kingdom.

We currently do not understand many aspects of human development. For instance, the process by which embryonic cells differentiate into specialised cell types remains largely a mystery. Recent evidence suggests that our current models, which are based on studies in mice, may be wrong.^22^ Using GGE to investigate the activity of particular genes in early human embryos would help unlock the secrets of early development. This could potentially lead to non‐GGE‐based clinical advances in the future – such as improving IVF success rates and reducing the incidence of miscarriage.^23^

GGE could also improve our understanding of genetic diseases. Embryonic stem cells altered to carry disease‐causing mutations can be used as models of genetic disease and substrates for in vitro drug testing. For example, embryonic stem cell lines could be created that carry the mutations associated with Parkinson's disease, and then induced to grow into nerve cells (which malfunction in Parkinson's disease). Potential drugs for Parkinson's disease could then be tested on these nerve cells. This could expedite the development of pharmacological treatments.

While such research currently takes place using induced pluripotent stem (IPS) cells, embryonic cells may have technical advantages.^24^ IPS cells are created from somatic cells, which may have undergone so‐called epigenetic changes. As a result, IPS cell lines are more diverse and behave less predictably than embryonic cell lines. Drug screening conducted on gene‐edited embryonic cells may lead to more reliable results.

Similar techniques could be used to generate stem cell therapies. Embryonic stem cells could be modified to carry alterations that provide protection against some disease. These cells could then be grown into various cell types for direct therapies. For example, gene‐edited embryonic cells could be used to create immune cells that target cancer. These modified cells could then be used as part of cancer treatments. Although using IPS cells in this way will be effective in some cases,^25^ embryonic cells contain fewer epigenetic changes and they could potentially have some unique applications.

The fact that the use of GGE in research could produce substantial benefits generates further *pro tanto* reasons in favour of allowing and funding some forms of germline gene editing. GGE could help us unlock the secrets of human development and the genesis of disease. Such knowledge may be valuable in its own right, in addition to leading to non‐GGE‐based treatments in the future.

In sum, there are significant reasons in favour of pursuing GGE. In the short term, GGE may be a valuable research tool and provide a novel treatment for single gene disorders. In the longer term, GGE could be an important tool in the fight against polygenic and infectious disease.

## 3 Objections to Pursuing GGE

In the above section, we argued that there is a significant case in favour of pursuing some types of GGE. In this section we analyse some of the arguments that have been offered against the pursuit of GGE to determine whether they undermine this case.

### 3.1 Safety

Many believe GGE research ought not to be permitted or funded because it is unsafe.

The National Institutes of Health (NIH) point to ‘serious and unquantifiable safety issues’ in justifying their negative stance toward GGE research.^26^ Concerns for safety were also prominent in commentaries which appeared in *Nature* and *Science* calling for GGE to be halted or strongly discouraged.^27^

The most obvious safety concerns regarding GGE stem from what are called ‘off‐target’ mutations – unintended changes to the genome. Off‐target mutations could potentially result in the development of cancer and other pathologies. Gene editing performed on human embryos that would subsequently be brought to term therefore risks causing disease and disability.

Are the safety risks associated with off‐target mutations strong enough to justify prohibiting or declining to fund all forms of GGE research? This is a familiar question. Nearly all medical research poses safety risks to participants. Safety risks are therefore routinely considered as part of research ethics frameworks.^28^ The first step in assessing safety risks is identifying who is at risk of harm. Thus, when assessing the risks of gene editing in human embryos, we should begin by asking who may be harmed by this research.

Some will say that the embryo itself is at risk of harm. But it is doubtful that the embryo is the type of entity that can be harmed, or at least, harmed in a morally significant way. The embryo does not have experiences or desires, and on some accounts of wellbeing, entities that lack experiences and desires have no wellbeing and thus cannot be harmed. Moreover, even if embryos can be harmed, it is doubtful whether harms to embryos have enough moral significance to justify prohibition or non‐funding of otherwise valuable research. Indeed, many jurisdictions currently permit and fund embryo research that involves certain destruction of the embryo, and this is widely thought to be permissible. Similarly, many jurisdictions permit and fund abortion, and the discarding of unwanted IVF embryos. It is doubtful that one could hold these practices to be permissible – as many do – while holding that the death of even an early embryo counts as a morally weighty harm.

Finally, even if the death of an embryo does count as a morally weighty harm, this may not count decisively against pursuing GGE in all circumstances. This is because, if GGE is developed to the point that it becomes safe for therapeutic uses, it could be used as a replacement for PGD which itself often results in the destruction of excess embryos. Thus, development of GGE could potentially reduce the number of human embryo deaths that will occur in the future.^29^

It is harms to future people, not embryos that are the most plausible basis for objections to GGE. If the embryos used in this research are brought to term, the children who are born as a result of these technologies could develop cancer or other diseases as a result of off‐target mutations.

There is, however, an easy way to protect future people from these safety risks: ensure none come into existence. As long as GGE results in off target mutations, we can ensure that these safety risks are avoided by making sure that none of the embryos used in the research are allowed to develop to the point that they are the subjects of morally weighty harms.

The study by Liang and co‐authors shows how the safety risks of GGE can be negated. The study was not conducted on embryos that were destined to be born, or indeed even had the potential to be born. The researchers used triploid embryos – embryos that have an extra set of chromosomes. These embryos cannot survive pregnancy, and are normally spontaneously aborted. Trialling the CRISPR system in these embryos had no chance of resulting in a live birth. Although the study did find a high rate of off‐target mutations, these did not result in morally significant harm.

Regulations that govern embryo research in several countries that allow such research are already sufficient to protect future people from the safety risks of GGE research. The UK, for instance, has laws allowing limited forms of genetic research to be conducted, provided that the embryo is destroyed by 14 days and not implanted into a woman.^30^ Given this legal requirement, and assuming compliance with the law, no GGE research conducted in the UK risks harming any future child.

Some might worry that pursuing GGE research under such a regulatory system will create pressure to relax laws in the future, and will thus lead indirectly to objectionably harmful research. If the rate of off‐target mutations significantly improves, legislators may lift the 14‐day limit and allow edited embryos to be brought to term. If this happens, it is important that any initial clinical trials using GGE are conducted with a focus on harm minimisation.

Early gene therapy trials were conducted with an emphasis on participant consent. The most infamous case is that of Jesse Gelsinger. In that case a somatic cell gene therapy was developed for ornithine transcarbamylase deficiency, a disorder of nitrogen metabolism. The condition comes in two forms: mild, with normal life expectancy and management by diet, and severe, which is lethal in the first year. Researchers, acting on the advice of ethicists, decided to conduct the first trials in adults with the mild form of the disease as they were capable of consenting. Gelsinger consented at age 18 and died due to a catastrophic immune reaction. He would have had a normal life expectancy in the absence of the intervention.^31^

The trial should have been conducted in infants with the severe form of the disease, as this would have resulted in less expected harm.^32^ The same principle applies to any trial of GGE – the first trials ought to be conducted in diseases which are lethal soon after birth. The reason for this is if the GGE technique turns out to be lethal, little is lost because that individual had no hope of long‐term survival in any case. While it is not possible to obtain consent from infant participants (though their parents must consent), it is necessary to weigh the value of consent against minimising expected harm.^33^

Indeed, other safety measures could be used to minimise safety risks for edited embryos resulting in live births. Testing of embryos and foetuses could be performed to evaluate the effectiveness of GGE. For example, PGD could be performed, with whole genome analysis, at day 3–4, chorionic villus sampling (CVS) at 10 weeks, and structural ultrasound at 20 weeks. If evidence of off target mutations were found, termination of pregnancy could be performed.

Given the ready availability of safety measures, there seems little reason to suppose that pursuing some forms of GGE now will lead to so much unsafe use of the technology in the future that we should not pursue it now.

### 3.2 Germline Changes

A common objection that has been levelled against the pursuit of GGE is that altering the germline will have negative consequences for future generations.^34^ This objection can take two forms: either GGE will make unintended changes to the germline, through off‐target mutations, which will have negative effects on future generations; or the changes we intend to make to the germline will have harmful unforeseen consequences.

It is clear that off‐target mutations associated with GGE could be harmful to future generations. The accumulation of random mutations may slowly lead to increasing rates of disease. However, it is plausible that as GGE develops the rate of off‐target mutations will become negligible. The rates of off‐targets mutations in animal models have been declining rapidly, and such mutations are now considered ‘undetectable’ in some applications.^35^ In addition, there will be ways to estimate the risk from off‐target mutations, as discussed above. This will further reduce the risk to future generations.

Still, some may argue that even just a few germline changes could cause widespread harm to future generations. It is possible that these will be missed in any safety checks that are performed. However, it seems unlikely that a small number of germline mutations pose a serious enough risk to future generations that we ought not to pursue GGE. Mutations are constantly being introduced in the human germline and many human activities increase the rate at which they occur. For example, delaying paternity increases the number of mutations in sperm, which are then passed on to children in the next generation. Not many believe these additional mutations represent a morally weighty risk to future generations. If they did, this would arguably justify screening the sperm of older fathers for mutations, or providing incentives for young men to freeze sperm for use later in life.^36^ Similarly, some cancer treatments potentially cause germline mutations.^37^ However not many argue that these mutations cause a weighty enough risk to future generations that we ought to screen the gametes of cancer survivors for mutations before allowing or assisting them to reproduce.

Therefore, if GGE will be deployed only at the point where the number of off‐target mutations is very small, the risk of harm to future generations is unlikely to count decisively against pursuing it. This risk is likely outweighed by the potential benefits of GGE for future generations, discussed in Section 2.

A different concern about the effect of GGE on future generations centres on the intended, rather than the unintended, changes we will make to the germline. We may use GGE to increase the frequency of genes that are beneficial in one generation, yet these same genes may be harmful to future generations.

Some genes provide protection against certain diseases but increase susceptibility to others. For example, it is known that a variant of the DARC gene – which codes for an antigen found on red blood cells – provides protection against malaria. However this version of the gene also disposes people to be more susceptible to human immunodeficiency virus (HIV). Suppose that in a region where malaria is prevalent and HIV rare many parents use GGE to give their children forms of the gene that protect against malaria. Subsequent generations could then be decimated by HIV. Other immune genes have known benefits but may also have costs that are yet to be discovered. For example, the CCR5 gene codes for a type of receptor found on macrophages (a type of white blood cell), which are targeted by the HIV virus. One form of the CCR5 gene provides resistance to the HIV virus. However, given the important role played by macrophage receptors in fighting other infections, it is possible that individuals with this form of the gene will be more susceptible to other infectious agents that are yet to evolve. If we use GGE to introduce the HIV resistant version of the CCR5 gene, this may make future generations susceptible to a future plague.^38^

There is no doubt that such concerns need to be carefully considered before GGE is used in a clinical setting. Current decision‐makers need to consider the interests of future generations, and should not reduce valuable forms of diversity.^39^ But these concerns do not show that GGE will cause harms to future generations that outweigh its benefits. Rather they demonstrate the need to take care in deciding when and how to deploy the technology and to consider the likely future environment in which that individual will exist.

### 3.3 The Consent and Autonomy of Future Generations

Some arguments against GGE dispute the authority of current individuals to make decisions on behalf of future generations. In outlining their decisions to continue not to fund GGE research, the NIH pointed to the ‘ethical issues presented by altering the germline in a way that affects the next generation without their consent.’^40^ This argument is pursued less directly in the *Nature* commentary, which refers to the difficulty in obtaining ‘informed consent’ when calling for a moratorium on GGE.^41^

It is not made clear in either piece why the consent of future generations should be seen as vital for decisions involving GGE, but not for other major decisions with long term effects. We cannot obtain the consent of future generations for the development of powerful communication technologies – like smartphones – which will dramatically alter their lives. It would be absurd to claim that we shouldn't develop any risky novel technologies because it ‘affects the next generation without their consent’.

The central question with GGE, as with all interventions that create risks for individuals who cannot consent, is not whether the individuals who would be exposed to the risks would consent to them, but whether they will also (expectably) enjoy benefits that outweigh the risks.

Suppose first that our pursuit of GGE will affect what future people come into existence. Thus, the individuals who will bear the risks of GGE will also exist only because we pursued it. These individuals will enjoy existential benefit from our pursuit of GGE. In rare cases, individuals may also suffer harms from GGE that outweigh these benefits in the sense that we have stronger reason to avoid the harms than to produce the benefits. This would most plausibly be so if GGE causes side‐effects so severe as to make an individual's life not worth living. But provided GGE is sensibly regulated so as to mitigate risks, such cases will be extremely rare. It thus seems reasonable to expect that, collectively, the existential benefits will outweigh the risks.

Now suppose that the individuals who bear the risks of our pursuing GGE would exist even if we did not pursue it. In that case, they will not enjoy an existential benefit. However, many will enjoy concrete person‐affecting health benefits in the form of reduced (risk of) disease. It seems nearly universally accepted that we can benefit people by reducing rates of disease. (This is consistent with how we currently treat people who are unable to offer consent, such as those who are unconscious. We often take it for granted that improving the health of these individuals would benefit them.) Moreover, it is again plausible that, if GGE is sensibly regulated, these benefits will outweigh the risks.

Some believe the important issue is not the consent of future individuals, but rather their autonomy. Habermas believes that the accidental nature of our genetic make‐up is vital for our ability to live as autonomous agents. It is only by having genes given to us by chance, rather than design, that we can ‘grow from nature’ and live autonomously.^42^ When we alter the genes of future individuals, we thereby undermine their autonomy.^43^

Habermas's account relies on a distinction between social and natural influences. On his account, what is wrong about genetic modification is that social forces determine our characteristics. By editing the genes of our children we allow social values to determine their heredity, and deprive them of having a genome determined by nature. However, what Habermas's account fails to recognise is that social forces have been affecting our genome for generations. Social ideals of beauty influence mate choice, and thereby directly influence the genetic makeup of the next generation. In addition, many social and cultural developments have changed the human germline in distinctive ways. For example, the development of agriculture led to the widespread selection of genes that allow humans to digest starch and lactose. Our genome has already been influenced by our social life.

It might be argued that there is a morally significant difference between GGE and other social influences on heredity: GGE is *intended* to influence the genomes of future generations, whereas other social influences are not. It might thus be argued that GGE amounts to the domination, manipulation or control of future generations by present ones, and, on some accounts of autonomy, it thereby reduces their autonomy.^44^ However, many actions taken by the parents of young children also intentionally influence the lives of those children. They do not do so via altering their genes directly, but it is difficult to see why this should be morally significant; our genes are just one causal influence on our lives. Indeed, some social and environmental influences affect gene expression through epigenetic effects, and these changes may be passed on to the next generation.^45^ Parenting actions are not normally regarded as significant threats to autonomy merely because they involve the domination, manipulation or control of children by their parents – they are normally regarded as such only when and because parental actions severely constrain the future life choices of their children or harm them directly. In cases where parents act in ways that expand the future life choices of their children, as eliminating a disposition to disease would generally do, this autonomy‐enhancing effect is normally thought to outweigh any restriction on autonomy due to the presence of domination, manipulation or control. It is arguably the presence of disease and disorder, not gene editing to remove them, that presents the greatest threat to future autonomy.^46^

Thus, even if we accept that GGE *in one way* reduces the autonomy of future generations, we believe this will often be outweighed by its other autonomy‐increasing effects. Suppose we use GGE to edit out a gene that causes cystic fibrosis, and thereby prevent an individual from suffering this disease. This individual can now live without the fear of the consequences of cystic fibrosis, and will not be impeded by the physical limitations imposed by the disease. Removing this gene promotes the individual's autonomy, and it seems plausible to suppose that this effect outweighs any reduction in autonomy due to the domination, manipulation or control of the individual by prior generations.

### 3.4 Enhancement

One common concern about GGE is that it will be used a tool of human enhancement and not merely to prevent disease. GGE has much greater capacity to be used as a means of enhancement than conventional selection methods. This is because it can target a large number of genes simultaneously and could be used to insert genes that would not occur naturally. While genetic selection allows selection within the normal human range, gene editing would allow the enhancement of human capacities to supranormal levels.

Many believe that if GGE were used a tool of human enhancement, it could cause widespread social harm. This seems to motivate Marcy Darnovsky, of The Center for Genetics and Society, when she says that ‘creating genetically modified human beings could easily lead to new forms of inequality, discrimination and societal conflict.’^47^ It is difficult to see how using GE to avoid genetic *disease* could lead to any of these things.

We have argued elsewhere that arguments against human enhancement face conceptual challenges.^48^ There are several different ways to understand the term ‘enhancement’, which are often only imprecisely communicated by opponents of enhancement. No commonly offered definitions describe something clearly morally problematic. Further difficulties arise when considering how biological enhancement can be differentiated from non‐biological enhancements, which are nearly universally celebrated.

These issues have been analysed in great detail elsewhere,^49^ and we do not have space to explore them here.

However, suppose for the sake of argument that biological enhancement is universally problematic. It is doubtful that this would count decisively against permitting and funding the therapeutic use of GGE, or the continuation of GGE research.

Many medical technologies currently being used or developed for the treatment of disease could also be used as enhancements. Many of those who are against the use of these technologies for enhancement purposes are still in favour of pursuing their development and therapeutic uses. Lasik eye surgery, pre‐implantation genetic diagnosis, and plastic surgery can be used non‐therapeutically, but this fact is not considered to provide reasons to prohibit or restrict funding for their therapeutic uses. This is because regulatory tools can be used to limit enhancement uses to such a level that the moral costs of enhancing uses are outweighed by the benefits of therapeutic applications. There is little reason to suppose that the situation would be different for GGE.

Some will argue that the stakes are much higher with GGE than for these other technologies. Furthermore, some are sceptical that regulations could prevent GGE from being used as an enhancement. We concede that if the use of GGE to enhance traits poses a very significant moral risk, and regulations could not limit GGE to therapeutic uses, then there may be a reason to not develop GGE as a clinical tool.^50^ However, we are aware of no empirical support for the claim that regulations could not also limit GGE to therapeutic applications (as has been the case for PGD) or morally innocuous enhancement applications.

In sum, while some of the arguments presented in this section point to concerns about some specific forms of GGE, and suggest ways we should guide and regulate its development, none count decisively against the pursuit of GGE. As it is impossible to get consent from people who do not yet exist, we cannot reasonably evaluate GGE through the lens of consent from future generations. The risk of germline changes and human enhancement is not unique to GGE, and merely shows that we need to research GGE thoroughly, and carefully regulate any clinical applications. While initial research with GGE may uncover further costs association with its pursuit; at this point, the costs are not clearly high enough to establish that GGE should not even be provisionally pursued.

## Conclusion

In this article we have shown that GGE research can be conducted safely in ways that carry manageable and reasonable risks. This of course would be moot if the development of GGE carried no benefits. But we have shown that there is a significant medical case for pursuing GGE to combat single gene disorders and polygenic disorders and, importantly, a research case for pursuing this technology to better understand the genesis of disease. The moral case in favour of pursuing GGE is stronger than the case against. This suggests that pursuing GGE is both morally permissible and morally desirable.^51^

This has direct implications for current policy debates. Given some research with GGE is warranted, we should resist calls for an international ban on all GGE research. Such bans risk depriving us of valuable knowledge about human development, and may deprive future generations of novel disease treatments.

## Notes

1 Puping Liang *et al*., ‘CRISPR/Cas9‐mediated gene editing in human tripronuclear zygotes’, *Protein and Cell* 6,5 (2015): 363–372.

2 In April 2016, a second study to alter embryonic DNA was reported. Further gene editing research in human embryos has been approved by regulatory bodies in the UK. Ewen Callaway, ‘Second Chinese team reports gene editing in human embryos’, *Nature News* (2016): doi:10.1038/nature.2016.19718

3 Helen Shen, ‘First monkeys with customized mutations born’, *Nature News* (2014): doi: 10.1038/nature.2014.14611

4 Luhan Yang *et al*., ‘Genome‐wide inactivation of porcine endogenous retroviruses (PERVs)’, *Science* (2015): doi: 10.1126/science.aad1191

5 Qingjian Zou *et al*., ‘Generation of gene‐target dogs using CRISPR/Cas9 system’, *Journal of Molecular Cell Biology* (2015): doi: 10.1093/jmcb/mjv061

6 For the rest of this article when we use the acronym GGE – we are referring to *human* germline gene editing.

7
*International Summit on Human Gene Editing: A Global Discussion. Washington DC, 1‐3 December 2015* (Washington DC: National Academies Press). Available from: http://www.ncbi.nlm.nih.gov/books/NBK343651/ (Accessed 22 September 2016).

8 Sarah Chan *et al*., ‘Genome editing technologies and human germline genetic modification: The Hinxton Group consensus statement’, *The American Journal of Bioethics* 15,12 (2015): 42–7.

9 The March of Dimes Birth Defects Foundation, *March of Dimes Global Report on Birth Defects* (New York: White Plains, 2006); John Harris, ‘Germ line modification and the burden of human existence’, *The Cambridge Quarterly of Health Care Ethics* 25,1 (2016): 6–18.

10 BBC News, ‘US “will not fund research for modifying embryo DNA”’. Available at <http://www.bbc.co.uk/news/health-32530334> (Accessed 7 October 2016).

11 E. Lanphier *et al*., ‘Don't edit the human germ line’, *Nature* 519,7544 (2015): 410–411.

12 Human Fertilisation & Embryology Authority, ‘Fertility treatment in 2011′, p. 17. Available at http://www.hfea.gov.uk/docs/HFEA_Fertility_Trends_and_Figures_2011_-_Annual_Register_Report.pdf (Accessed 7 October 2016).

13 We assume here that GGE does not induce changes so significant as to alter the identity of the person to which the embryo will give rise.

14 See Jeff McMahan, ‘Causing people to exist and saving people's lives’, *Journal of Ethics* 17 (2013): 5–35; Julian Savulescu, ‘The nature of the moral obligation to select the best children’ in A. Akabayashi (ed.) *Future of Bioethics: International Dialogues* (Oxford: Oxford University Press, 2014), pp. 170–182.

15 Struan F.A Grant & Hakon Hakonarson, ‘Genome‐wide association studies in type 1 diabetes’, *Current Diabetes Reports* 9,2 (2009): 157–163.

16 J.F. Peden & M. Farrall, ‘Thirty‐five common variants for coronary artery disease: The fruits of much collaborative labour’, *Human Molecular Genetics* 20,R2 (2011): R198–R205.

17 Christine Q. Chang *et al*., ‘A systematic review of cancer GWAS and candidate gene meta‐analyses reveals limited overlap but similar effect sizes’, *European Journal of Human Genetics* 22,3 (2013): 402–408.

18 Hannah Bourne,Thomas Douglas & Julian Savulescu, ‘Procreative beneficence and in vitro gametogenesis’, *Monash Bioethics Review* 30,2 (2012): 29–48.

19 Xiangjin Kang *et al*., ‘Introducing precise genetic modifications into human 3PN embryos by CRISPR/Cas‐mediated genome editing’, *Journal of Assisted Reproduction and Genetics* 33,5 (2016): 581–8.

20 Angela L. Rasmussen *et al*., ‘Host genetic diversity enables Ebola hemorrhagic fever pathogenesis and resistance’, *Science* 346,6212 (2014): 987–91.

21 M. Tabebordbar *et al*., ‘In vivo gene editing in dystrophic mouse muscle and muscle stem cells’, *Science* 351,6271 (2016): 407–11.

22 For example, see Naoko Irie *et al*., ‘SOX17 is a critical specifier of human primordial germ cell fate’, *Cell* 160,1–2 (2015): 253–68.

23 D. Mathews, S. Chan, P. Donovan, T. Douglas, C. Gyngell, J. Harris, A. Regenberg & R. Lovell‐Badge, ‘CRISPR: A path through the thicket’, *Nature*, 527,7577 (2015): 159–161.

24 Kazim H Narsinh, Jordan Plews & Joseph C. Wu, ‘Comparison of human induced pluripotent and embryonic stem cells: Fraternal or identical twins?’, *Molecular Therapy* 19,4 (2011): 635–8; Josipa Bilic & Juan Carlos Izpisua Belmonte, ‘Concise review: Induced pluripotent stem cells versus embryonic stem cells: close enough or yet too far apart?’, *Stem Cells* 30,1 (2012): 33–41.

25 Marina Riera *et al*., ‘Comparative study of human embryonic stem cells (hESC) and human induced pluripotent stem cells (hiPSC) as a treatment for retinal dystrophies’, *Molecular Therapy* – *Methods & Clinical Development* 3 (2016): 16010.

26 Francis Collins, ‘Statement on NIH funding of research using gene‐editing technologies in human embryos’, 2015. Available at http://www.nih.gov/about/director/04292015_statement_gene_editing_technologies.htm (Accessed 7 October 2016).

27 Lanphier *et al*. op. cit.; D. Baltimore *et al*., ‘A prudent path forward for genomic engineering and germline gene modification’, *Science* 348,6230 (2015): 36–38.

28 S. Shaw, ‘Research governance: regulating risk and reducing harm?’, *Journal of the Royal Society of Medicine* 99,1 (2006): 14–19.

29 Julian Savulescu, ‘The embryonic stem cell lottery and the cannibalization of human beings’, *Bioethics* 16,6 (2002): 508–529. Whether this counts in favour of GGE will depend on how we understand the supposed moral prohibition on killing human embryos. If we understand this as a deontological side‐constraint, then it may not be possible to justify the killing of embryos now to avoid a greater number of embryo deaths in the future. By contrast, if the prohibition is an implication of a consequence‐based duty to prevent harm, then the prevention of future embryo deaths could justify the causing of embryo deaths now.

30 The Human Fertilisation and Embryology Authority, *Human Fertilisation and Embryology Act 2008*. Available at: http://www.legislation.gov.uk/ukpga/2008/22/pdfs/ukpga_20080022_en.pdf (Accessed 7 October 2016).

31 J. Kaiser, ‘Gene therapy: Questions remain on cause of death in arthritis trial’, *Science* 317,5845 (2007): 1665a.

32 Julian Savulescu, ‘Harm, ethics committees and the gene therapy death,’ *Journal of Medical Ethics* 27 (2001): 148–150.

33 We discuss issues of consent further in Section 3.3.

34 Lanphier *et al*. op. cit.

35 Benjamin P. Kleinstiver *et al*., ‘High‐fidelity CRISPR–Cas9 nucleases with no detectable genome‐wide off‐target effects’, *Nature* 529,7587 (2016): 490–5.

36 Although this has been suggested that such acts would be justified, See Kevin R Smith, ‘Paternal age bioethics’, *Journal of Medical Ethics* 41,9 (2015): 775–779.

37 C.D. Glen & Y.E. Dubrova, ‘Exposure to anticancer drugs can result in transgenerational genomic instability in mice’, *Proceedings of the National Academy of Sciences* 109,8 (2012): 2984–8.

38 Chris Gyngell & Thomas Douglas, ‘Stocking the genetic supermarket: Reproductive genetic technologies and collective action problems’, *Bioethics* 29,4 (2015): 241–250.

39 Ibid.

40 Collins op. cit.

41 Lanphier *et al*. op. cit.

42 Jürgen Habermas, *The Future of Human Nature* (Cambridge: Polity, 2003).

43 For a critical discussion of Habermas's account see: Jonathan Pugh, ‘Autonomy, natality and freedom: A liberal re‐examination of Habermas in the enhancement debate’, *Bioethics* 29,3 (2014): 145–152.

44 Robert Sparrow, ‘Better living through chemistry? A reply to Savulescu and Persson on “Moral Enhancement”’, *Journal of Applied Philosophy* 31,1 (2014): 23–32.

45 M.J Meaney, ‘Maternal care, gene expression, and the transmission of individual differences in stress reactivity across generations,’ *Annual Review of Neuroscience* 24 (2011): 1161–1192.

46 We are assuming here that our pursuit of GGE would not be identity‐affecting for those whose autonomy would allegedly be constrained. If it would, then the putative constraint on autonomy would need to be weighed against an existential benefit, and again, it is plausible that the benefit would be weightier in almost all cases.

47 The Center for Genetics and Society, ‘Public interest group calls for strengthening global policies against human germline modification’ [online]. Available at: http://www.geneticsandsociety.org/article.php?id=8528 (Accessed 7 October 2016).

48 J. Savulescu, A. Sandberg & G. Kahane, ‘Well‐being and enhancement’, in J. Savulescu, R. ter Meulen & G. Kahane (eds) *Enhancing Human Capacities* (Oxford: Wiley‐Blackwell, 2011), pp. 3–18; Thomas Douglas, ‘Enhancement, biomedical’ in H. LaFollette (ed.) *International Encyclopedia of Ethics* (Oxford: Wiley‐Blackwell, 2013); B. Earp, A. Sandberg, G. Kahane & J. Savulescu, ‘When is diminishment a form of enhancement? Rethinking the enhancement debate in biomedical ethics’, *Frontiers in Systems Neuroscience* 8,12 (2014): 1–7; C. Gyngell & M. Selgelid, ‘Human enhancement: Conceptual clarity and moral significance’ in S.Clarke, A Giubilini, J. Savulescu, T. Coady & S. Sanyal (eds) *The Ethics of Human Enhancement: Understanding the Debate* (Oxford: Oxford University Press, 2016), pp. 111–126.

49 See Allen Buchanan, Dan Brock, Norman Daniels & Daniel Wikler, *From Chance To Choice* (Cambridge: Cambridge University Press, 2000); Leon Kass, *Beyond Therapy* (New York: Dana Press, 2003); John Harris, *Enhancing Evolution* (Princeton, NJ: Princeton University Press, 2007); Julian Savulescu & Nick Bostrom, *Human Enhancement* (Oxford: Oxford University Press, 2009).

50 Note, though, even this wouldn't be a reason against the use of GGE in research.

51 It may also imply that pursuing GGE is morally *obligatory*, for instance, because we are morally obliged to do what we have most moral reason to do. However, we do not argue for this claim here.

